# Fine-Scale Quantification of Absorbed Photosynthetically Active Radiation (APAR) in Plantation Forests with 3D Radiative Transfer Modeling and LiDAR Data

**DOI:** 10.34133/plantphenomics.0166

**Published:** 2024-04-08

**Authors:** Xun Zhao, Jianbo Qi, Zhexiu Yu, Lijuan Yuan, Huaguo Huang

**Affiliations:** ^1^ Key Laboratory for Silviculture and Conservation of Ministry of Education, Beijing Forestry University, Beijing 100083, China.; ^2^ Innovation Research Center of Satellite Application, Faculty of Geographical Science, Beijing Normal University, Beijing 100875, China.

## Abstract

Quantifying the relationship between light and stands or individual trees is of great significance in understanding tree competition, improving forest productivity, and comprehending ecological processes. However, accurately depicting the spatiotemporal variability of light under complex forest structural conditions poses a challenge, especially for precise forest management decisions that require a quantitative study of the relationship between fine-scale individual tree structure and light. 3D RTMs (3-dimensional radiative transfer models), which accurately characterize the interaction between solar radiation and detailed forest scenes, provide a reliable means for depicting such relationships. This study employs a 3D RTM and LiDAR (light detection and ranging) data to characterize the light environment of larch plantations at a fine spatiotemporal scale, further investigating the relationship between absorbed photosynthetically active radiation (APAR) and forest structures. The impact of specific tree structural parameters, such as crown diameter, crown area, crown length, crown ratio, crown volume, tree height, leaf area index, and a distance parameter assessing tree competition, on the daily-scale cumulative APAR per tree was investigated using a partial least squares regression (PLSR) model. Furthermore, variable importance in projection (VIP) was also calculated from the PLSR. The results indicate that among the individual tree structure parameters, crown volume is the most important one in explaining individual tree APAR (VIP = 4.19), while the competition from surrounding trees still plays a role in explaining individual tree APAR to some extent (VIP = 0.15), and crown ratio contributes the least (VIP = 0.03). Regarding the spatial distribution of trees, the average cumulative APAR per tree of larch plots does not increase with an increase in canopy gap fraction. Tree density and average cumulative APAR per tree were fitted using a natural exponential equation, with a coefficient of determination (*R*^2^ = 0.89), and a small mean absolute percentage error (MAPE = 0.03). This study demonstrates the potential of combining 3D RTM with LiDAR data to quantify fine-scale APAR in plantations, providing insights for optimizing forest structure, enhancing forest quality, and implementing precise forest management practices, such as selective breeding for superior tree species.

## Introduction

As one of the primary components of terrestrial ecosystems, plantation forests not only provide goods for social development but also play a crucial role in regulating global climate and maintaining the balance of carbon cycling [1]. China’s plantation forest area of 69.33 million hectares ranks first in the world, accounting for 36% of the total forest area in China [2]. In order to enhance forest carbon sequestration and address environmental conservation needs, China will continue to expand the plantation forest area. It is projected that by 2050, the forest coverage area will be increased to 26% [3]. However, compared to natural forests, certain plantation forests, such as larch plantations, exhibit characteristics of poor quality, low productivity, and low carbon storage [4]. Therefore, as the expansion of plantation forests continues, it becomes increasingly urgent to improve forest productivity and enhance forest carbon storage.

Among the numerous factors influencing vegetation growth, such as light, water, and temperature, light is the primary driver of photosynthesis in plants, substantially influencing forest development and tree growth [5,6]. In terms of the light, the photosynthetically active radiation (PAR) energy absorbed by vegetation within the 400- to 700-nm spectral range serves as a key variable for estimating vegetation ecosystem productivity [7]. However, the complex canopy structure of forests exhibits high 3D heterogeneity, leading to variations in the distribution of light environments within forest, which further affects forest productivity [8], resistance to disturbances [9], forest species diversity [10], and carbon storage capacity [11]. Therefore, accurate characterization of forest absorbed photosynthetically active radiation (APAR) contributes to insights into achieving sustainable development of plantation forests and improving forest productivity.

Noticeable variations in light interception occur among different stands, tree species, and even individual trees, leading to productivity differences within the forest. The forest canopy is the part of trees that directly responds to solar radiation during photosynthesis. However, due to the complexity of the forest canopy, such as tree spatial arrangement and canopy structure, it influences the local distribution of light environment within the forest [12]. Several studies have indicated that canopy elements such as branches and leaves are the main factors affecting light interception during canopy photosynthesis [13,14]. Additionally, the spatial distribution of tree canopies also affects the light use efficiency of stands, further influencing stand productivity variations [15]. Therefore, it is essential to quantify the relationship between light and forest structure in order to facilitate precise forest management.

Due to the spatiotemporal variability of light, measuring the direct impact of the canopy structure on light interception is difficult. Therefore, precise characterization of the complex relationship between forest structure and light has also been a major challenge in understanding forest productivity and ecological functionality [16]. Traditional approaches mainly rely on quantum sensors and optical devices for direct measurements in the field [17]. However, these direct measurement methods are time-consuming and labor-intensive, and face challenges in characterizing the spatiotemporal continuous distribution of light within the forest canopy [18]. To overcome these challenges, some modeling approaches have also been used to describe the distribution of light within the canopy [19,20]. Among them, the Beer–Lambert law [21] is the most widely used model, assuming the canopy as a turbid medium with a single layer. However, for the heterogeneity of the forest canopy, it is difficult for the model to accurately capture the fine-scale distribution of light within the canopy [22]. Furthermore, algorithms based on remote sensing data have been developed to provide an effective approach for acquiring spatiotemporal continuous information on light. The MODIS (moderate-resolution imaging spectrometer) satellite, utilizing the theory of spectral invariant, is employed to retrieve FPAR (fraction of photosynthetically active radiation) and LAI (leaf area index) products [23,24]. At the same time, some researchers have also established empirical relationships between remote sensing vegetation indices and PAR [25,26].However, all the methods mentioned above, whether employing optical devices or quantum sensors for direct field measurements of light distribution or using simple physical, empirical or semi-empirical models, are unable to finely describe light distribution within canopy. Particularly, quantifying light at the tree level poses significant challenges.

Therefore, conducting complex forest light distribution simulations based on detailed forest scenes is a reliable approach. With the advancement of current computer technology, some 3-dimensional radiative transfer models (3D RTMs) based on explicitly described scenes have emerged, such as DART (discrete anisotropic radiative transfer) [27] and LESS (large-scale remote sensing data and image simulation framework) [28,29]. These models have been widely used for remote sensing algorithm evaluation, validation, and development [30,31] due to their ability to accurately characterize the interaction between complex vegetation structures and solar radiation. Therefore, 3D RTMs have been used for studying the terrain surface radiation budget [32]. Meanwhile, with the increasing availability and low cost of acquiring light detection and ranging (LiDAR) data, as well as the ability to quantitatively and accurately characterize complex forest structures [33], LiDAR data have become the primary input source for current 3D RTMs [34]. Previous studies have also utilized LiDAR data combined with a 3D RTM to simulate the temporal and spatial variations of light within different complex forest structures at daily or seasonal scales [16,35]. However, these studies still focus on the plot level, lacking sufficient understanding of key tree structure indicators, the spatial distribution of trees, and their impact on light interception. These limitations also hinder the precise management of forests.

In this study, we primarily utilize LiDAR data combined with a 3D radiative transfer model LESS [28,29] to conduct fine-scale individual tree-level APAR simulations in larch plantations. Our objective is to conduct a detailed investigation of light interception by individual trees combining a 3D RTM, which has the capability to accurately characterize forest canopies and solar radiation interactions, with high-resolution LiDAR data that allow for quantitative analysis of tree structure and spatial distribution. Specifically, our main objective is to answer the following questions: How does one quantitatively and accurately assess the relationship between individual tree structure and APAR? How does the spatial distribution of trees affect APAR? Based on these relationships, a convenient and effective technical means is provided to enhance forest quality, optimize forest structure, and facilitate precise forest management.

## Materials and Methods

### Study area and plot data collection

#### Study area

**Fig. 1.**
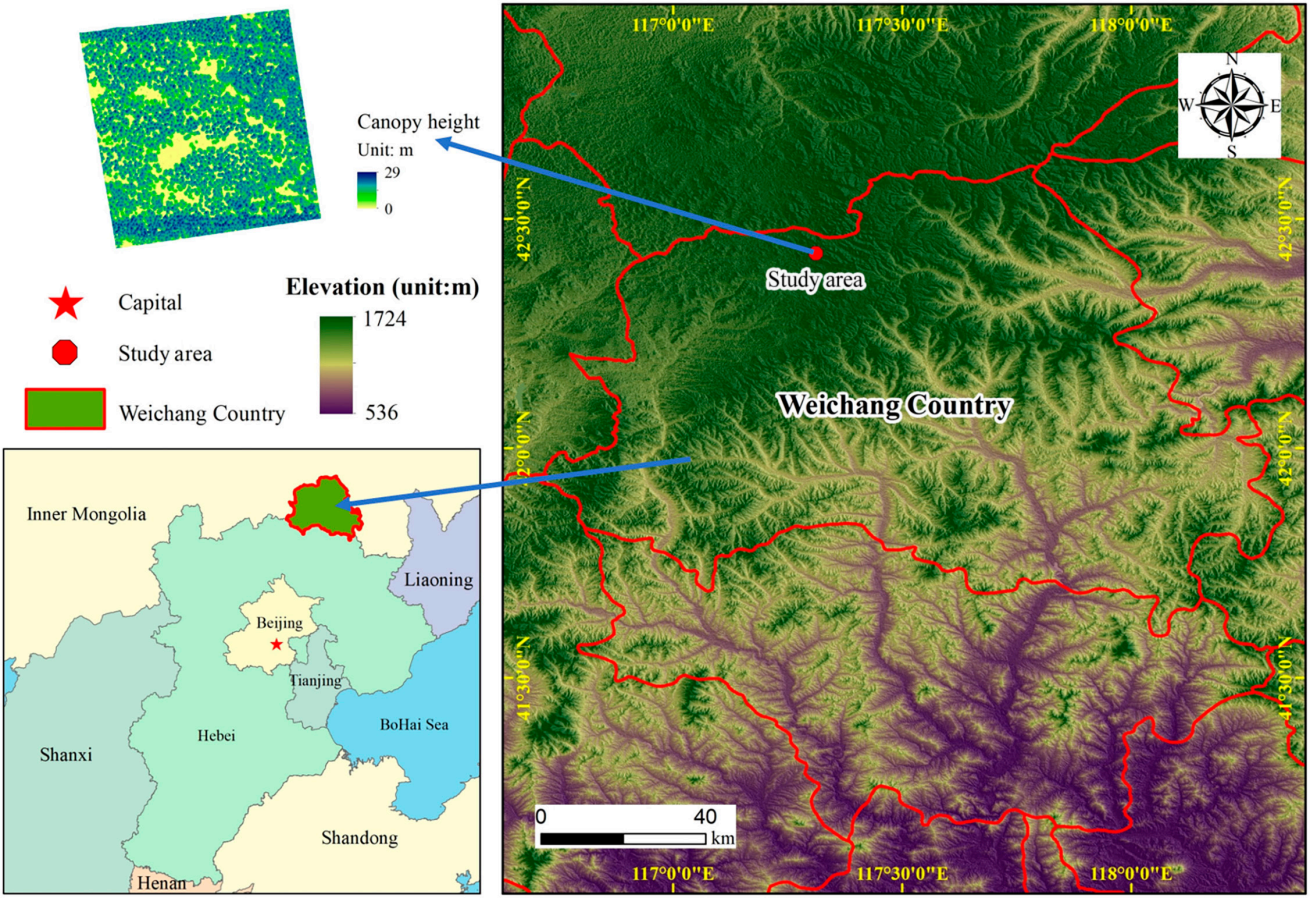
Research area location diagram.

Our study area is a 300 × 300 m plot located within a Larix zone of the Saihanba Forest Farm (SFF) (Fig. 1), which is situated in Weichang County, Hebei Province in northern China. The SFF has been awarded the “LAND FOR LIFE AWARD” by the United Nations and is the largest larch plantation in China, covering a total area of approximately 72,000 hectares. The dominant tree species in the area is larch (*Larix principis-rupprechtii Mayr*). Currently, larch accounts for over 70% of the forest area in SFF. It is a fast-growing native coniferous tree species with beneficial green economic value. Therefore, it plays a vital role in promoting ecological and social benefits in northern China.

#### Plot data collection

On 2022 July 21, we employed the RIEGL VUX-1UAV LiDAR sensor mounted on a drone to obtain fine-grained structural data of a larch forest in the research area. The entire larch forest (300 m × 300 m) was scanned from a flight height of 60 m. The acquired point cloud density was approximately 548 points/m^2^. After completing the scanning process, we utilized digital hemispherical photography to capture hemispherical images of the larch forest. Based on the acquired digital hemispherical photography, a CLX algorithm [36] was employed to calculate the LAI of the plots, resulting in an approximate value of 3.3. The corresponding needle spectra, bark spectra, and soil spectra were also measured using an ASD spectrometer (FieldSpec®4 Hi-Res NG) after completing the digital hemispherical photography measurements. Furthermore, to avoid interference from leaf point cloud data in reconstructing the fine-grained details of stem and branch structures, we conducted terrestrial laser scanning (TLS) during the leaf-off period of the previous year. This scanning covered a 50 m × 50 m area of deciduous pine forest near the study area. This TLS was carried out using the RIEGL VZ-1000, with horizontal and vertical scanning angles of 0.03°.

### Processing of airborne LiDAR data

**Fig. 2.**
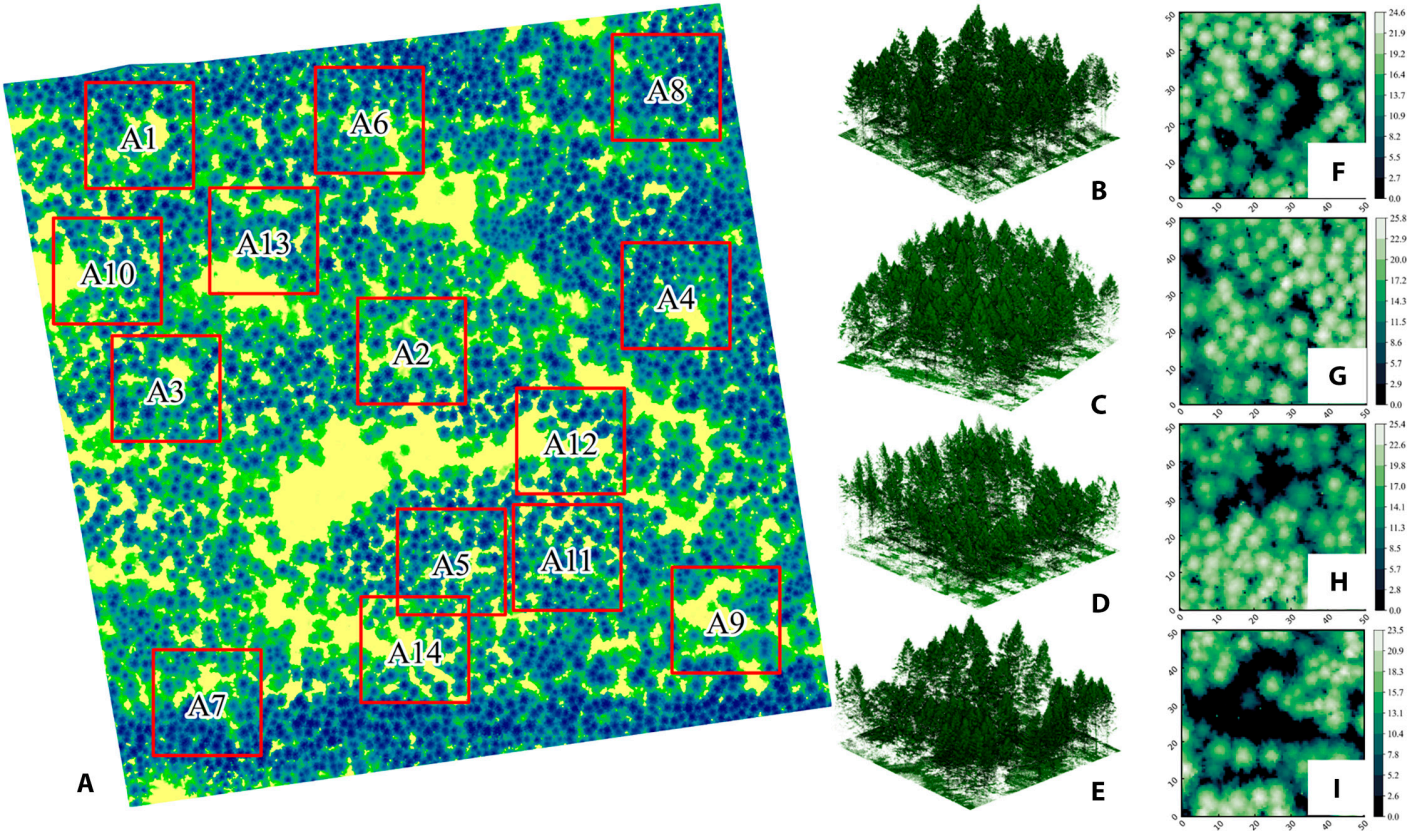
The distribution of the 14 larch plots and the normalized point cloud data along with the corresponding canopy height models (CHMs) for some typical plots. The red borders represent the boundaries of the 14 larch plots (A). The normalized point cloud data for each larch plot in the upper, lower, left, and right areas (A1, A7, A8, and A9) (B to E) within the study area, along with the corresponding CHMs (F to I) for each plot.

Realistic larch structural parameters, including tree positions, heights, and crown areas, are acquired by airborne LiDAR, providing the foundational tree structure and distribution parameters for the construction of a detailed 3D virtual larch plot. We first select 9 larch plots (50 m × 50 m) with a uniform sampling approach within the 300 m × 300 m study area. To enhance the robustness of the sampling, an additional 5 larch plots were also randomly chosen. In total, 14 larch plots were used to quantify the relationship between light and structure (Fig. 2A). The spatial vector data of defined 14 larch plots was used to clip the airborne point clouds of the larch plots. Noise points were removed from the airborne point cloud data of 14 larch plots using the statistical outlier removal algorithm [37], which calculated the average distance and standard deviation between each point and its nearest 20 points. Each point exceeding the threshold of average distance plus 5 times the standard deviation was identified as a noise point and subsequently removed. The denoised point cloud data of the 14 larch plots were classified into ground and non-ground categories by the CSF (cloth simulation filtering) algorithm [38] to achieve the normalization of point cloud data for each plot. Furthermore, the normalized point cloud data (Fig. 2B to E) of the larch plots were rasterized into 0.5-m grid images to generate canopy height models (CHMs) (Fig. 2F to I), and any unnatural pits were smoothed out by the pit-free algorithm [39] in the CHM for the accurate segmentation of individual trees. Tree segmentation was performed based on the smoothed CHM data of the 14 larch plots. Specifically, a circular window with a diameter of 3 m was first applied to the CHM of each larch plot, and local maxima within the window were extracted as the tree top points of the current tree crown. Additionally, to eliminate the influence of understory shrubs, we excluded tree top points with a height below 2 m. Finally, utilizing the combination of detected tree top points and CHM, an individual tree segmentation algorithm [40] was employed to accomplish the segmentation of individual trees for the 14 larch plots. Furthermore, tree height, crown area, and tree location (*X*, *Y*) for the 14 larch plots were obtained. The aforementioned point cloud processing steps and tree segmentation were performed using the lidR software [41].

### Construction of the larch single-tree database

**Fig. 3.**
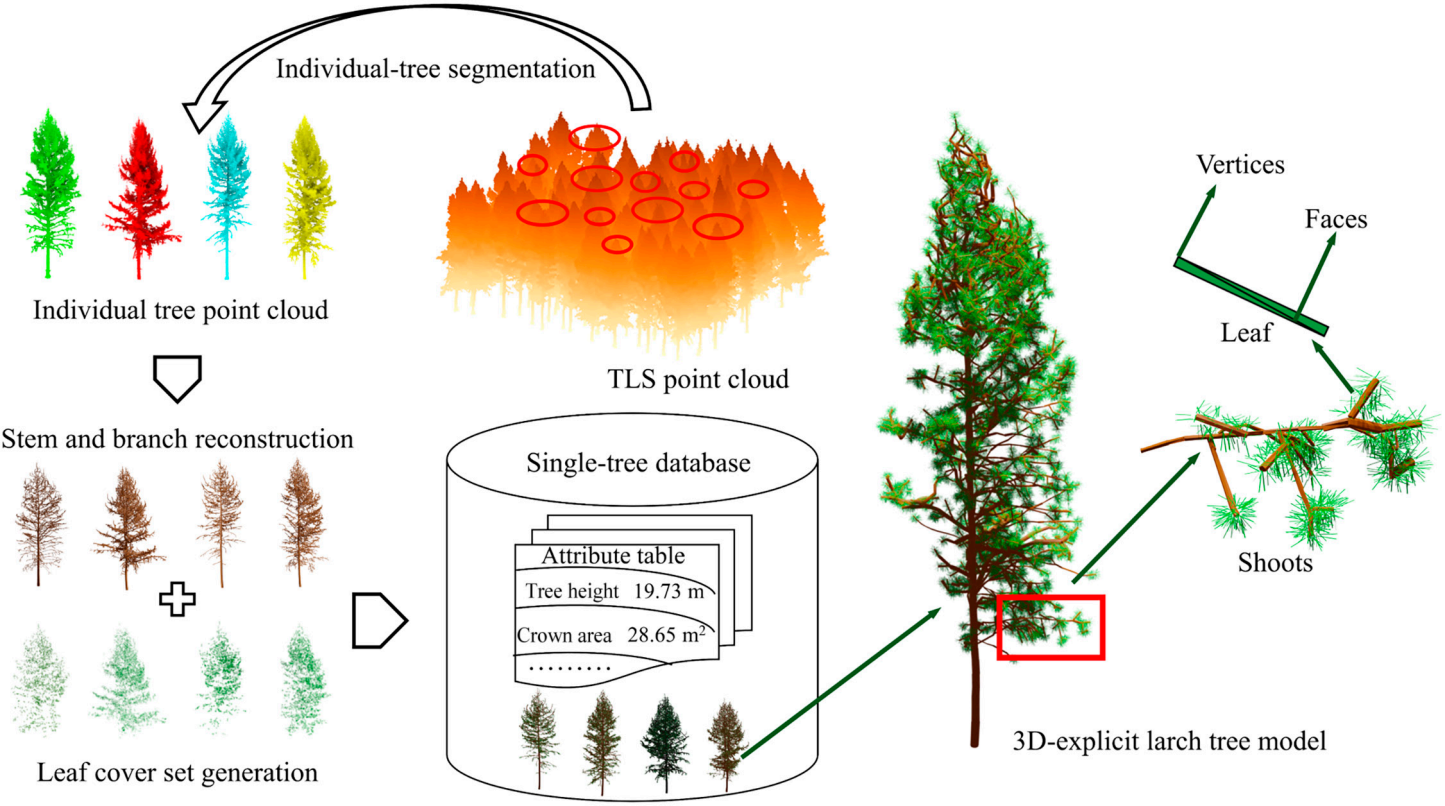
Generation of an explicit database of individual larch trees.

The larch single-tree database will be reconstructed based on TLS data to provide a foundational geometric tree model repository for constructing each 3D virtual larch plot. To reconstruct the detailed model of larch stems and branches, interactive manual segmentation of the point cloud data of individual larch trees from TLS measurement was initially performed using CloudCompare software. During the segmentation process, to ensure a high-quality reconstruction, individual trees within incomplete point cloud due to mutual occlusion were removed for reconstruction. The remaining individual tree point cloud data were used to reconstruct 3D-explicit tree models. Specifically, the TreeQSM algorithm [42] was utilized to fit the point cloud data of each individual tree’s stems and branches, thereby generating quantitative structure models (QSMs). Leaves were then added based on the QSM structure to generate explicitly 3D tree model (Fig. 3). However, the shape of the leaf was simplified to balance the computational resources consumed by the 3D RTM. Specifically, a rectangular solid facet (Fig. 3), composed of 4 vertices and 2 triangular faces, was inserted at the tips of the branches using the FaNNI [43] algorithm. A uniform sampling of leaf size with length range (6.5 to 8.5 cm) similar to the real leaf was also performed to ensure the realism of needle size. The total leaf area of each reconstructed individual tree is controlled. Specifically, the total leaf area of the plot was obtained by multiplying the measured LAI by the reconstructed plot area (50 m × 50 m). The total leaf area of the plot is then divided by the total number of larch trees in the entire plot to represent the total leaf area (LA) of each individual tree [34]. Finally, a 3D tree model is generated for each larch tree in the single-tree database. In addition, several tree structural parameters, such as crown area, crown volume, crown diameter, crown length, tree height, crown ratio (ratio of the crown length to the tree height), and LAI (ratio of the LA to the crown area) are also exported from each reconstructed 3D tree model to generate the attribute table for the larch single-tree database (Fig. 3).

### Generation of virtual larch plots

**Fig. 4.**
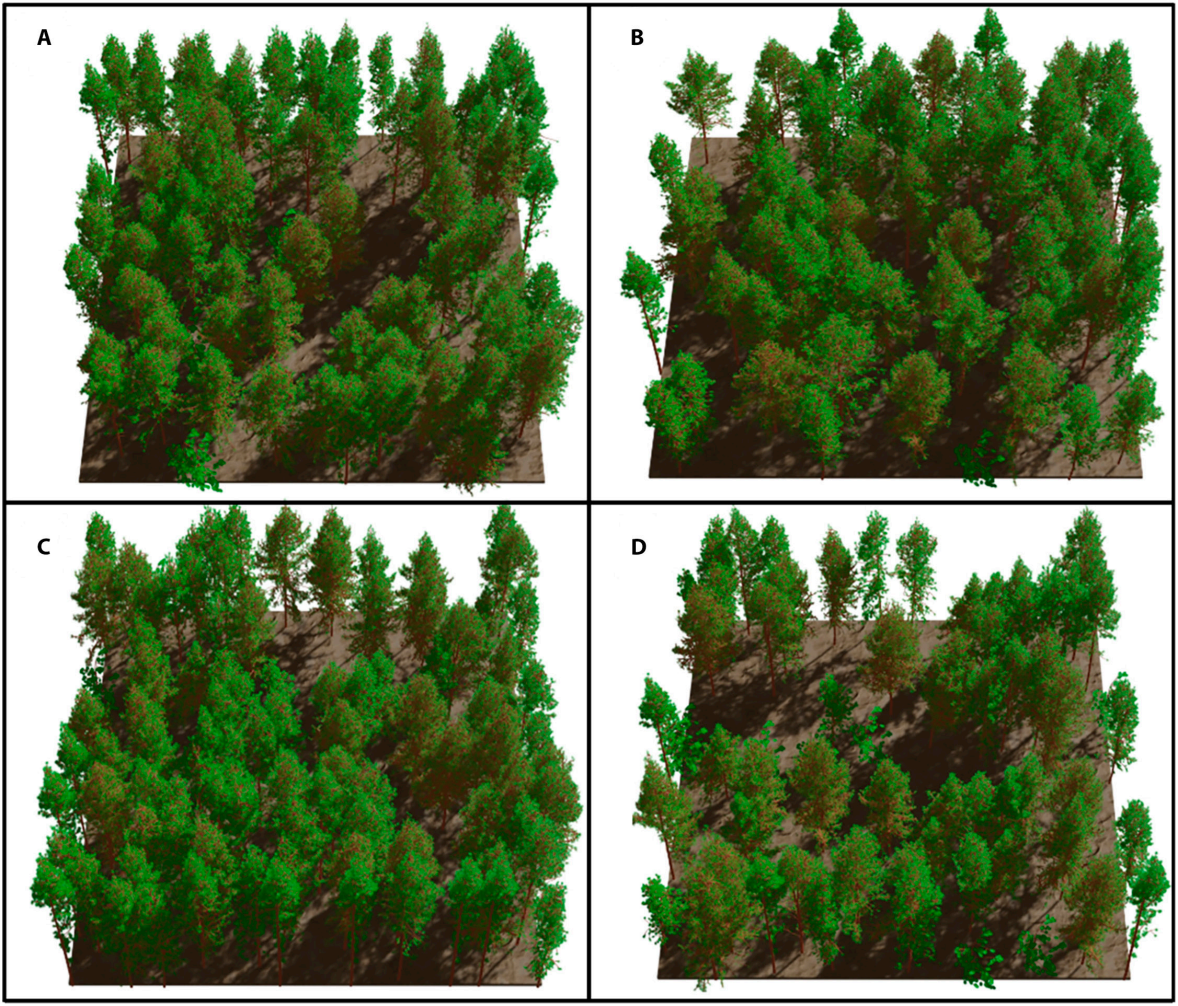
The generated 4 typical larch plots are presented in (A) to (D), labeled as A1, A8, A7, and A9 with reconstructed explicit tree structures.

Based on the foundational data (“Processing of airborne LiDAR data” section) of larch tree positions, heights, and crown area parameters obtained from airborne LiDAR, these data are utilized to query a single-tree database (“Construction of the larch single-tree database” section) obtained from TLS LiDAR. Single-tree structures are then placed at their corresponding positions to construct 14 virtual 3D larch scenes similar to real larch plots. We primarily referred to a method proposed by Qi et al. [44] for constructing large-scale forest scenes (1 km × 1 km) to generate realistic 3D virtual larch scenes. The foundational parameters needed by the reconstruction of the virtual larch plots was formed by the coordinates (*X*, *Y*), crown area, and height of individual trees obtained through airborne LiDAR. The reconstructed individual tree database was then searched using the crown area and tree height derived from the airborne point cloud at each detected tree position to find the best match. Specifically, for each tree position, the attribute table of the single-tree database was iterated through, and the sum of differences between the tree height and crown area derived from the airborne LiDAR and the corresponding reconstructed tree model’s tree height and crown area was calculated. The tree model with the minimum sum of differences was then placed at the actual position (*X*, *Y*) of the plot. Finally, virtual larch plots were generated (Fig. 4). Meanwhile, the individual tree structural parameters corresponding to 14 larch plots were also exported from the single-tree database to investigate the relationship between tree structure and APAR.

### Parameterization of 3-dimensional radiative transfer model

**Fig. 5.**
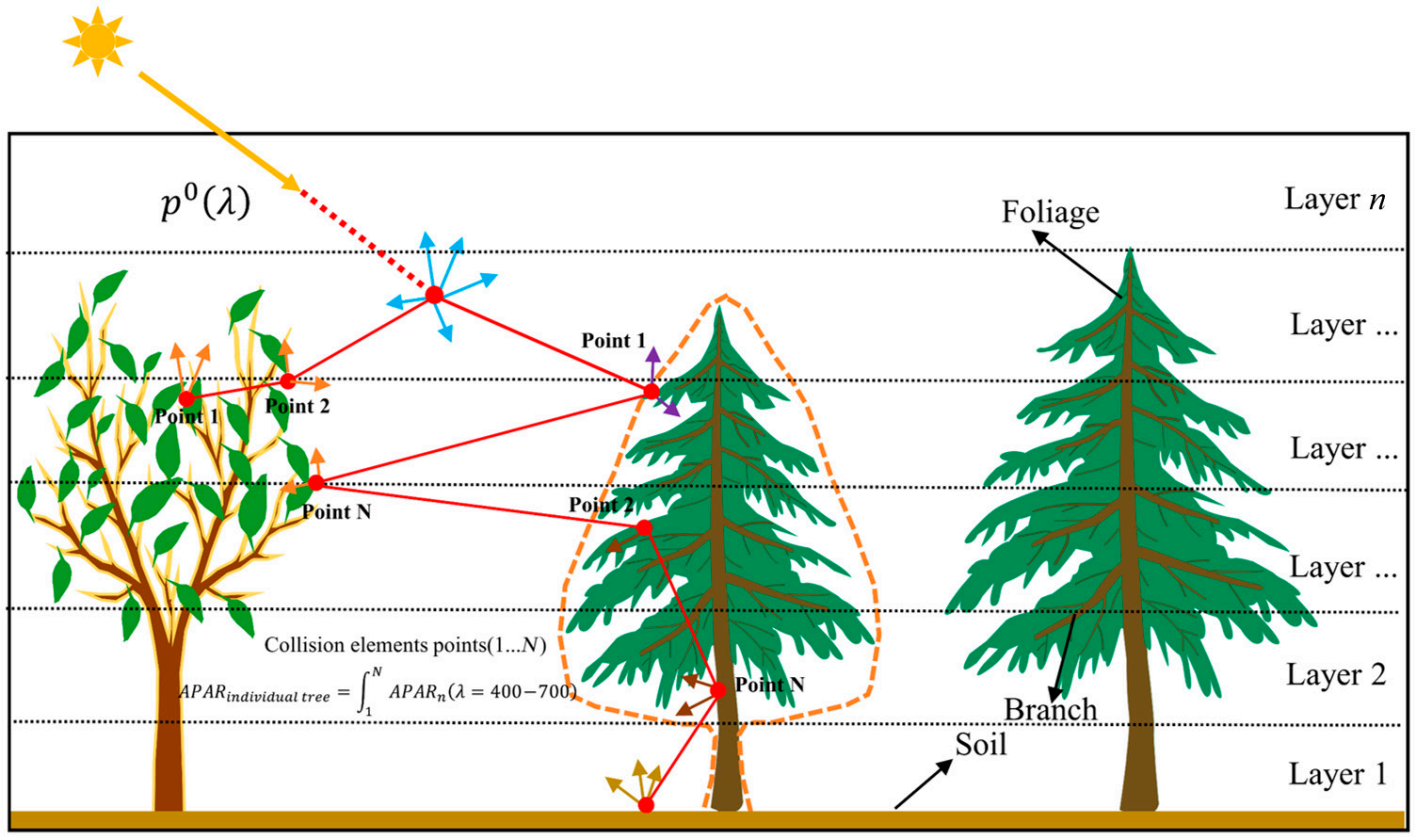
Schematic diagram of the APAR simulation principle at individual tree scale.

LESS [29] is a ray-tracing-based 3D RTM that offers both forward and backward ray tracing modes for different simulation tasks, and users can achieve precise simulation of remote sensing signals in arbitrarily complex scenes. The model also achieves high efficiency in signal simulation through parallel computing. To facilitate the model’s usage and batch simulation of remote sensing signals, LESS also provides a user-friendly graphical user interface and an easy-to-use batch processing Python SDK interface to meet users’simulation needs. Simultaneously, it has been validated against the reference benchmark scenes provided by the International Radiation Transfer Model Initiative [45], demonstrating results that are comparable in accuracy and reliability to those of DART. LESS has also been applied for the algorithm validation of the retrieval of green vegetation fraction absorbed photosynthetically active radiation (FAPAR_green_) [46], the evaluation of MODIS satellite LAI/FPAR algorithms [47], and the quantitative analysis of solar radiation over snow-covered mountains in the southeastern Tibetan Plateau [48]. In LESS, the simulation of APAR is primarily carried out through the forward ray tracing method. This involves calculating the collision points between photons and elements (e.g., leaves) in the scene, as well as considering the optical properties of these elements (Fig. 5). Specifically, when a photon with an initial energy of *p*^0^(*λ*) incident to the scene, the scattered energy *p^Q^* of Lambertian surfaces after *Q* times of collisions will be:

$$p^{Q}\left(\lambda \right)=p^{0}\left(\lambda \right)\prod_{q=1}^{Q}\left(x_{q,\lambda }/p_{q}\right)$$ (1)

where *x*_*q*,*λ*_ is either the reflectance *x*_*q*,*λ*_ or transmittance *τ*_*q*,*λ*_ of the collision surface according to a probability of *p_q_*. Forcefully choosing either a reflectance or transmittance according to a user-defined probability of *p_q_* (e.g., 0.5) is to avoid splitting a photon into a reflect photon and a transmitted photon to save ray intersection time [29]. Then at the *Q*th collision, the total APAR at this point can be computed used using Eq. 2. Furthermore, based on the location of the photon collision or the type of element collided, LESS will then accumulate the *APAR*_*λ*=400−700_ for each facet, layer, or individual object (e.g., branch or foliage) within the scene.

$${\text{APAR}}_{\lambda =400-700}=\int_{400}^{700}p^{Q-1}\left(\lambda \right)\left(1-x_{q,\lambda }/p_{q}\right)\mathrm{d\lambda}$$ (2)

where *APAR*_*λ*=400−700_ stands for the total APAR within a wavelength range of 400 to 700 nm.

In this study, LESS v2.2.1 was employed to simulate fine-scale individual tree APAR simulation in larch stands at a daily scale with a 2-h interval (8:00 to 18:00). Specifically, APAR simulations were conducted within the spectral range of 400 to 700 nm with a spectral resolution of 6 nm. To account for atmospheric effects in APAR simulations, the 6S atmospheric model was used to calculate sky scattering and direct solar radiation at 6 time points throughout the day for 14 larch plots based on mid-latitude summer atmospheric profile and elevation data from the study area’s center points. These calculated sky scattering and direct solar radiation were then used as input parameters in the LESS model to simulate diurnal individual tree APAR for each larch plot. In order to reduce edge effects, the scene surrounding the 50 m × 50 m grid of larch forest was replicated in each direction (up, down, left, and right), approximating the creation of a larger forest by the LESS model. The 4-component images of the 14 larch plots were also simulated using the LESS model to calculate the canopy gap fraction of the each virtual larch plot, aiming to investigate its relationship with APAR in larch plots. In total, the model was run 84 times for simulations (6 time points ×14 plots).

### Relationship between tree parameters and daily-scale cumulative APAR

#### Individual tree parameters vs. APAR

The partial least squares regression (PLSR) model was employed to inventory the relationship between the individual tree structure and diurnal cumulative APAR per tree in the 14 virtual larch plots. Specifically, the total amount of instantaneous individual tree APAR was calculated at 6 time points within a day, which was then integrated as the daily-scale cumulative APAR per tree. For the quantification of individual tree structures, the corresponding individual tree structural parameters were extracted from the single-tree database for the 14 virtual larch plots. These parameters included tree height, crown area, crown diameter, crown volume, crown length, crown ratio, and LAI. At the same time, we also consider the influence of neighboring trees on the target tree in a day. Previous studies have shown that neighboring trees within a certain radius around the target tree are prone to compete for light, water, temperature, or other nutrients, which can have an impact on the target tree [49,50]. The size of the search radius is usually determined in conjunction with factors such as the crown size. For instance, some studies use a fixed radius of 10 m around the target tree to assess the effects of neighboring trees on target tree in coniferous forests [51,52]. Therefore, we iterated each individual tree in each larch plot as a target tree and calculated the average distance of the surrounding neighboring trees within a 10-m radius as a measure of the influence factor of the surrounding neighboring trees on the target tree. Finally, the relationship was established by combining the individual tree structural parameters, a distance-dependent competition index (CI), and the daily-scale cumulative APAR per tree using the PLSR model. Among them, 70% of the individual tree data from the 14 virtual larch plots were used as training samples to train the PLSR model, while the remaining 30% of the data were used as validation data to test the model. Root mean square error (RMSE), coefficient of determination (*R*^2^), and mean absolute percentage error (MAPE) were also used as 3 statistical metrics to assess the accuracy of the model. Variable importance in projection (VIP) scores were also obtained from the PLSR model to quantify the contribution of individual tree structural parameters to light interception [53].

#### Individual tree positions vs. APAR

To quantify the influence of tree spatial arrangement on daily-scale average cumulative APAR per tree in larch plots, we calculated the average cumulative APAR per tree in the entire larch plot. This was done by dividing the total APAR absorbed by all individual trees in each larch plot by the total number of trees to represent the daily-scale average cumulative APAR per tree for each larch plot. Stand density and canopy gap fraction were used as the main indicators to quantify the forest spatial arrangement for the 14 virtual larch plots. The forest stand density is derived from the individual tree counts of 14 virtual larch plots, which are further converted into tree density per hectare. Canopy gap fraction refers to the average values of the illuminated soil band and shaded soil band derived from LESS simulated 4-component images corresponding to each scene. In this foundation, we employed a fitting equation of the natural exponential function to fit the tree density and daily-scale average cumulative APAR of the 14 larch plots.

## Results

### The distribution of APAR within a day in larch forests

**Fig. 6.**
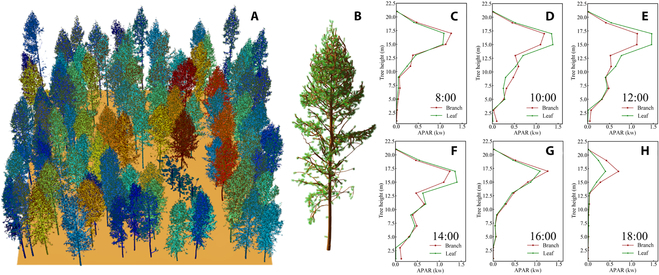
The simulated 3D distribution of individual tree APAR in the larch plantation. The 3-dimensional distribution of individual tree ARAR in plot A4 at 12:00 (A). The vertical light distribution of an individual tree (B) in plot A4 throughout the day from 8:00 to 18:00 is shown in (C) to (H).

**Fig. 7.**
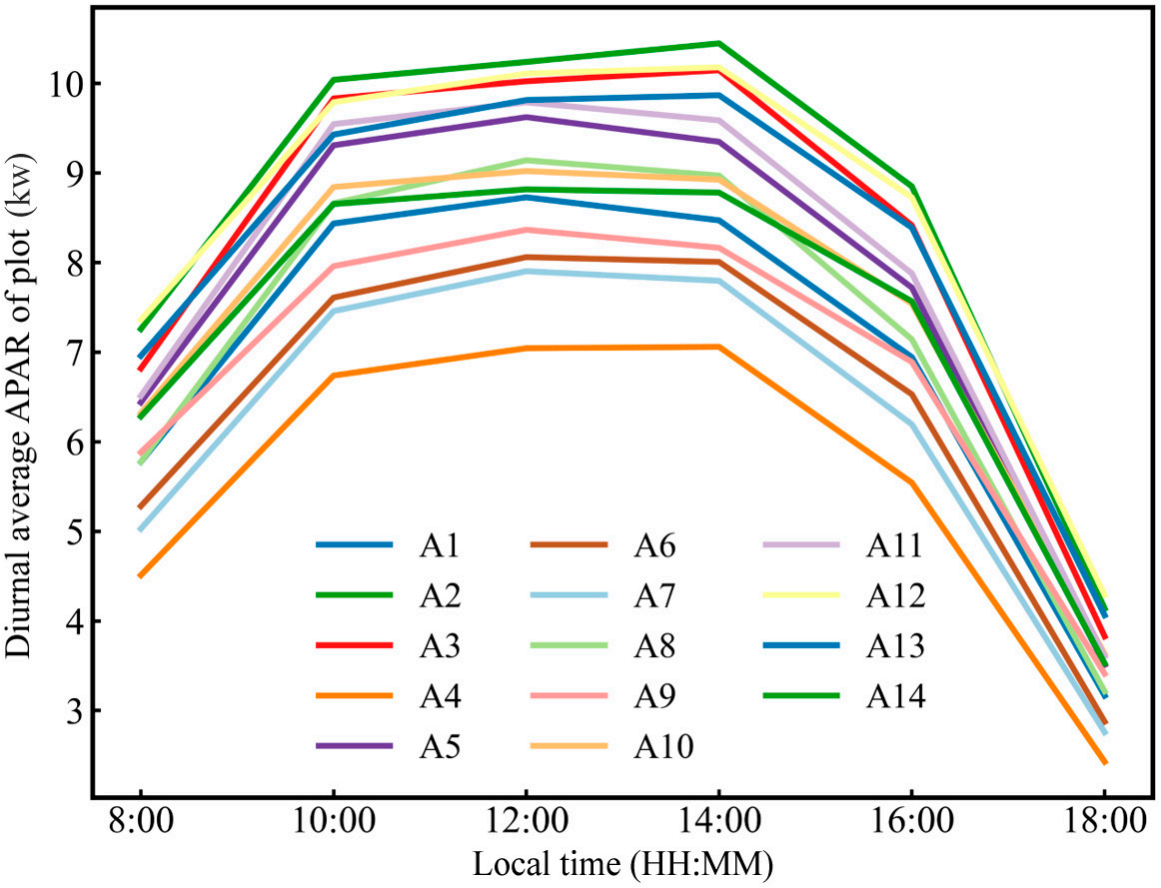
The average instantaneous APAR per tree of 14 virtual larch plots at different time points within a day.

**Fig. 8.**
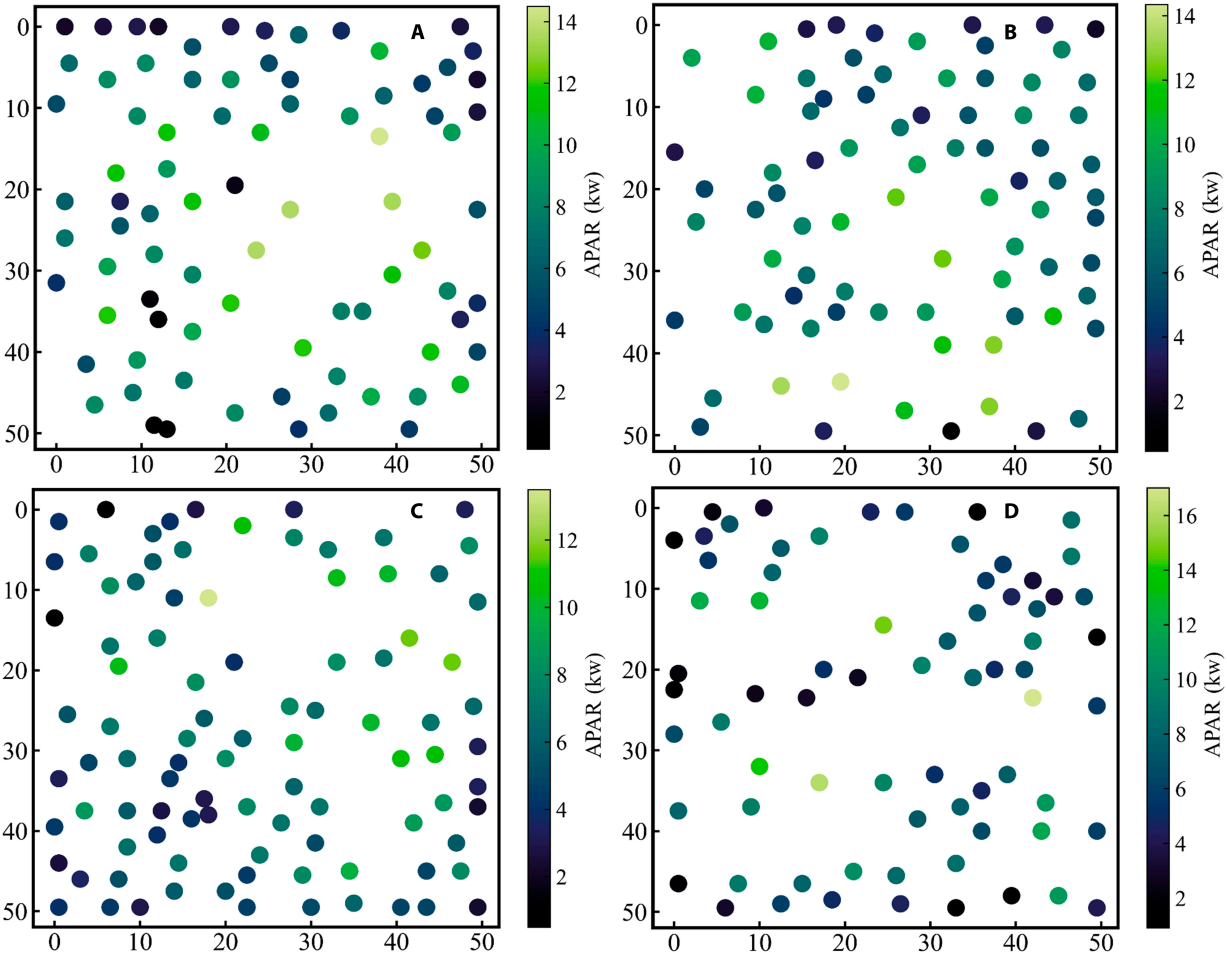
The spatial distribution maps of the cumulative mean APAR per tree for the 4 typical larch plots. The corresponding plot numbers are labeled as A1, A8, A7, and A9 (A to D).

The 3-dimensional distribution of APAR in a simulated larch plantation is presented in Fig. 6A. It can be observed that there is a 3D distribution of APAR among different individual trees. Within the forest plot, we can arbitrarily choose an individual tree (Fig. 6B). Furthermore, the vertical light distribution corresponding to each selected tree at 6 different time points (8:00 to 18:00) throughout the day can also be clearly depicted in Fig. 6C to H. The average instantaneous APAR per tree of each corresponding plot at 6 time points within a day is presented in Fig. 7, which shows an increasing trend followed by a decreasing trend within a day. During the time period of 12:00 to 14:00, the average instantaneous APAR per tree of all 14 larch plots reaches its peak; this can be attributed to the relatively high solar altitude angle at these time points, which were 66.72° and 59.44°, respectively. With the increase of solar altitude angle, the irradiance per unit area within the plot increased, resulting in the 12:00 and 14:00 time points exhibiting the highest distribution of average instantaneous APAR per tree among the 6 time points throughout the day. The spatial distribution map of the cumulative mean APAR per tree was also generated to visualize the mean amount of APAR by individual larch trees within a day for larch plots (Fig. 8).

### Daily-scale cumulative APAR model of individual tree

**Fig. 9.**
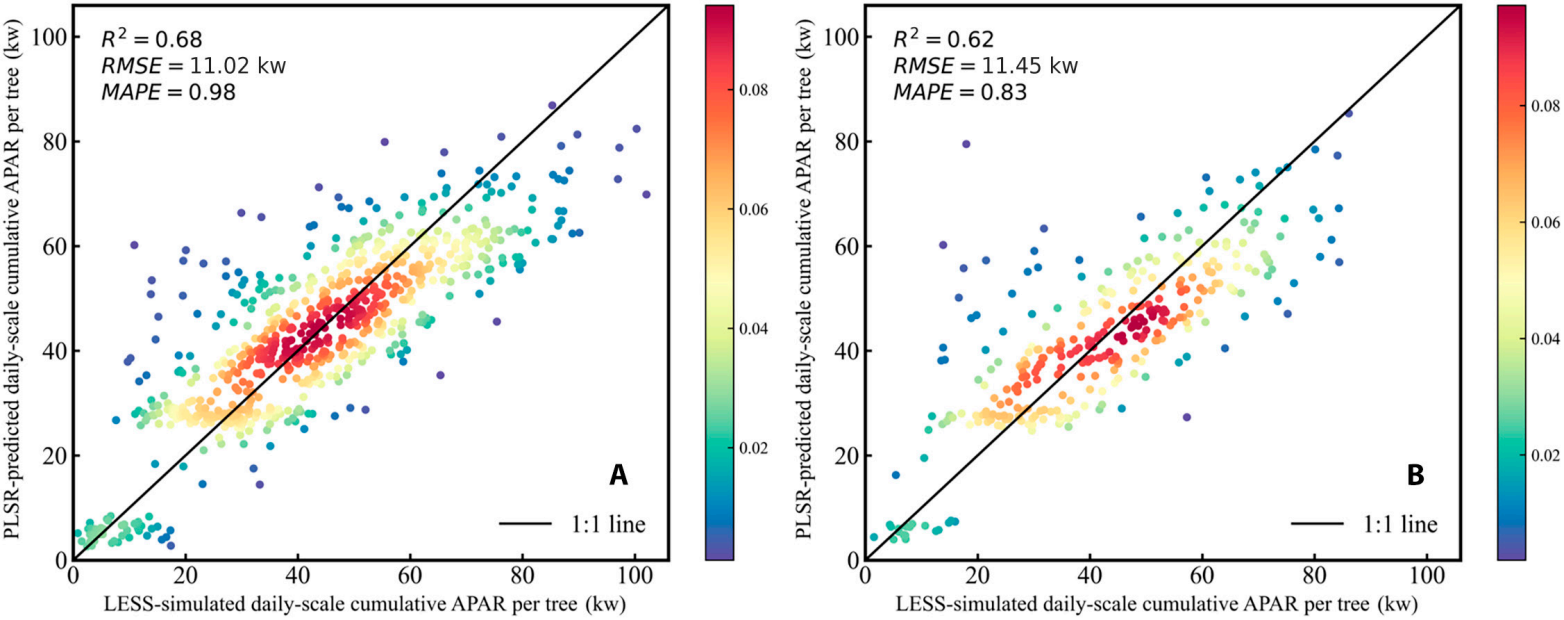
Comparison between PLSR model predictions of daily-scale cumulative APAR per tree and LESS simulated daily-scale cumulative APAR per tree. (A) The PLSR model was trained using a 70% subset of individual tree data. (B) The PLSR model was tested using a 30% subset of individual tree data.

The simulated APAR dataset of individual trees from the 14 virtual larch plots was first randomly divided into a 70% training set and a 30% test set for the establishment and validation of the PLSR model. Specifically, individual tree structural parameters (tree height, crown area, crown diameter, crown volume, crown length, crown ratio, and LAI) were obtained from the single-tree database as independent variables, while the daily-scale cumulative APAR per tree was used as the dependent variable to inventory the relationship between individual tree structure and daily-scale cumulative APAR per tree using the PLSR model. The comparison between LESS-simulated APAR and PLSR model-predicted APAR for the 70% training set and 30% test set is presented in Fig. 9. In the 70% training set (Fig. 9A), the *R*^2^, RMSE, and MAPE of the PLSR model were 0.68, 11.02 kw, and 0.98, respectively. In the 30% test set (Fig. 9B), the *R*^2^, RMSE, and MAPE of the PLSR model were 0.62, 11.45 kw, and 0.83, respectively. In addition, the VIP scores for each independent variable were also calculated from the PLSR model to quantify the importance of individual tree structure on the daily-scale cumulative APAR per tree. The individual tree structure variables corresponding to the VIP scores are presented in Table. It can be observed that the crown volume of individual trees has the highest importance on intercepting light (VIP = 4.19), followed by crown area (VIP = 1.37), and crown ratio has the least contribution (VIP = 0.03).

**Table.** Partial least squares regression (PLSR) model and corresponding variable importance in projection (VIP) scores of individual tree structural parameters. a, b, c, d, e, f, g, and h are the coefficients of the PLSR model.

Partial least squares regression (PLSR)	APAR = a × TH + b × CD + c × CL + d × CV + e × CA + f × CR + g × LAI + h × CI							
Independent variable (IV)	Tree height (TH)	Crown diameter (CD)	Crown length (CL)	Crown volume (CV)	Crown area (CA)	Crown ratio (CR)	Leaf area index (LAI)	Distance-dependent competition index (CI)
Variable importance in projection (VIP)	0.53	0.46	0.55	4.19	1.37	0.03	0.51	0.15

### Relationship between tree spatial arrangement and average cumulative APAR

**Fig. 10.**
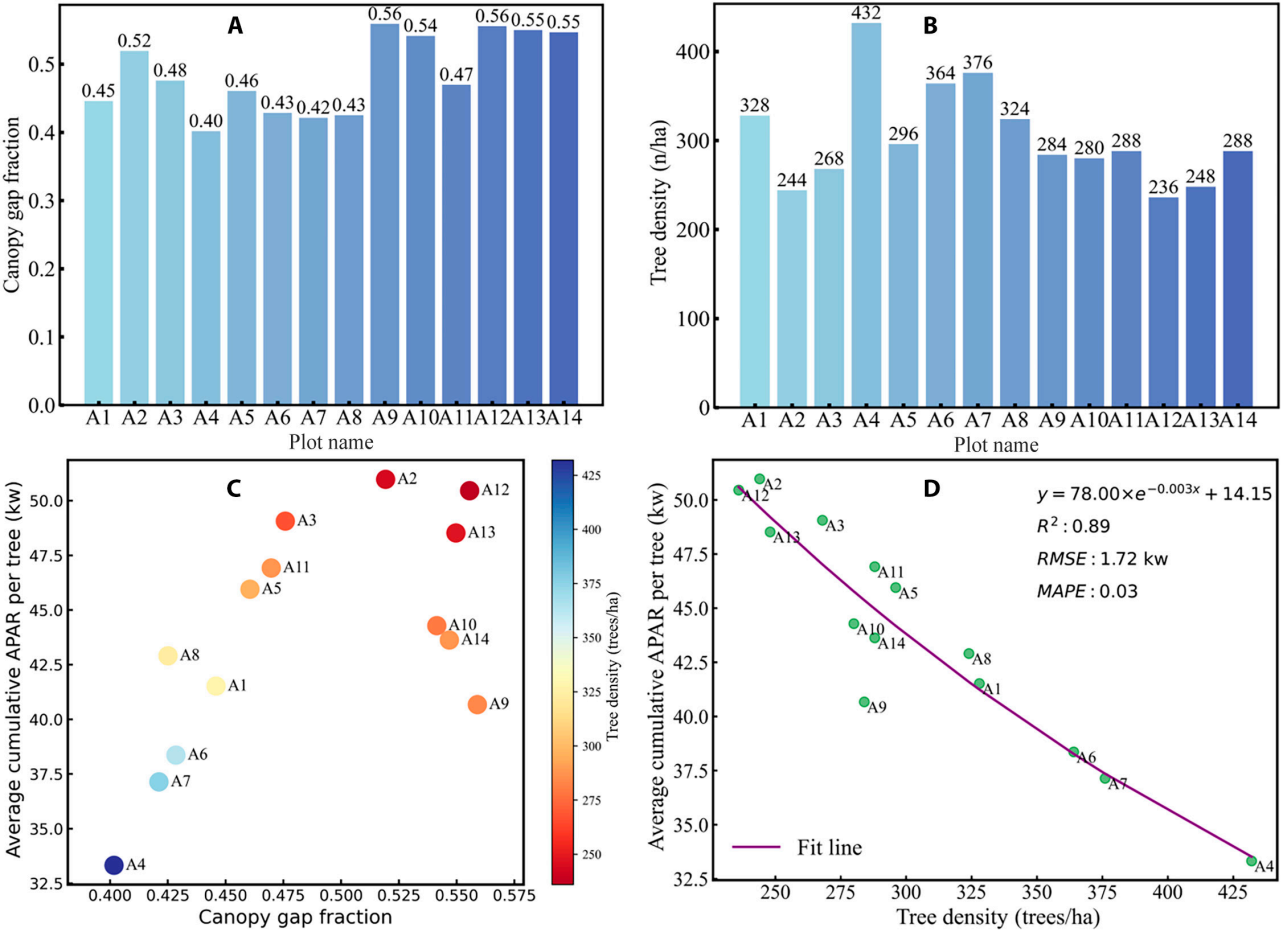
The canopy gap fraction and stand density of 14 virtual larch scenes, along with their relationships with the average cumulative APAR per tree. Tree density (A); canopy gap fraction (B). The canopy gap fractions were sorted in ascending order, and then the relationship among canopy gap fraction, average cumulative APAR per tree, and tree density was plotted (C). The tree density of the 14 larch plots was sorted in ascending order, and then the average cumulative APAR per tree and tree density were fitted using a natural exponential function (D).

Based on the stand density (Fig. 10A) and canopy gap fraction (Fig. 10B) obtained from the 14 virtual larch plots as described in the “Individual tree positions vs. APAR” section, the relationship between tree spatial arrangement and average cumulative APAR per tree was also quantified in Fig. 10C and D. Specifically, based on the ascending order of canopy gap fraction values, the relationship between sorted canopy gap fraction, corresponding stand density, and average cumulative APAR per tree was presented in Fig. 10C. It can be observed that the 14 larch plots show an initial increasing trend followed by a decreasing trend in average cumulative APAR per tree as the canopy gap fraction increases. The plots reach their peak average cumulative APAR per tree when the canopy gap fraction reaches 0.52 (plot A2). Furthermore, to elucidate the relationship between stand density structure and average cumulative APAR per tree, the tree densities of the 14 larch plots were also sorted in ascending order. The sorted tree densities and their corresponding average cumulative APAR per tree were fitted using a natural exponential function (Fig. 10D). It can be observed that the average cumulative APAR per tree and tree density can be well fitted by a natural exponential function, with a determination coefficient (*R*^2^) of 0.89, an RMSE of 1.72 kw, and a MAPE of 0.03.

## Discussion

### Generation of 3-dimensional forest virtual scenes

In this study, we combined 3D-explicit virtual larch scenes with a state-of-the-art 3D radiative transfer model called LESS to quantitatively investigate the relationship between light and forest structures. This approach helps us conduct lightweight, controllable simulation experiments of forest management, providing valuable insights into practical aspects such as optimal tree density selection (Fig. 10D). It is evident that the virtual 3D forest scene serves as a crucial foundation for representing the authenticity of the physical world in an abstract form, allowing us to characterize the relationship between light and forest structure through a 3D RTM. However, compared to the abstract representations of tree crowns using methods such as ellipsoid, cones, alpha shapes, and voxels [28], our primary approach involves generating 3D tree models with explicitly defined geometric structures of trees using high-precision LiDAR data. In addition, we fully utilize the advantages of ground-based and airborne LiDAR. Compared to airborne LiDAR, ground-based LiDAR scans smaller areas (plot scales), but it provides more precise 3D branch structure information [54]. Therefore, we employ ground-based LiDAR for reconstructing 3D tree models, and the structural parameters of these reconstructed models can be quantified using the QSM. On the other hand, airborne LiDAR has the capability to acquire data over larger regions and provides attributes of real physical forests, such as tree height, crown area, and position [55]. The combined advantages of ground-based and airborne LiDAR lay the foundation for constructing forest scenes at a large regional scale. Similar forest scene reconstruction approaches have also been found in the research conducted by Qi et al. [44], further demonstrating the potential for utilizing such methods in remote sensing image simulation and forest management studies. Visual comparisons were made between the reconstructed 14 virtual forest plots and the CHMs derived from airborne LiDAR data, demonstrating a high level of consistency with actual forest scenes. In addition, based on the real tree height and crown area parameters obtained from airborne LiDAR, we conducted a search in the single-tree database to place trees in their respective positions within the scene in order to achieve a spatially explicit representation of the larch forest. The tree height (RMSE = 1.12 m) and crown area (RMSE = 1.44 m^2^) of each placed 3D tree model showed significance agreement with the values obtained from airborne LiDAR for 14 larch forests.

### The importance of individual tree structures on light interception

The shape, size, dimensions, and leaf area of individual tree have significant effects on light interception [56]. In this study, several individual tree structural parameters (e.g., crown diameter, crown area, crown length, crown ratio, crown volume, tree height, and LAI) were extracted from the 3D-explicit tree model. A PLSR model was employed to quantitatively assess the relationship between forest tree structural parameters and daily-scale cumulative APAR per tree. The VIP scores calculated from the PLSR model indicate the contributions of different tree structural parameters to light interception (Table). Among the height-related individual tree structural parameters, the VIP scores for crown ratio, tree height, and crown length are 0.03, 0.53, and 0.55, respectively. The horizontally related individual tree structural parameters, including crown diameter, LAI, and crown area, have VIP scores of 0.46, 0.51, and 1.37, respectively. The VIP score for crown volume is 4.19, which measures the ecological niche space occupied by the entire tree crown in the stand. Furthermore, neighboring trees also have a certain impact on the light interception of the target tree itself (VIP = 0.15). The overall contribution to light interception shows that the height-related structural parameters (crown ratio, tree height, and crown length) have a lower impact compared to the horizontally related individual tree structural parameters (crown diameter, LAI, and crown area), while the parameter representing the spatial occupation of the tree crown (crown volume) has the highest contribution. The main reason for this is due to the relationship between light attenuation and the depth of the forest canopy. According to Beer–Lambert’s law, as the forest canopy becomes deeper, light exponentially decreases from top to bottom within the canopy [57]. Therefore, height-related structural parameters have a smaller contribution to light interception, with crown ratio having the smallest contribution. The contribution of horizontally related structural parameters to light interception lies between the two. This is mainly because light penetrates the canopy surface, and the overall size of the tree crown on a horizontal level, such as crown diameter, crown area, and the total distribution of leaf area across the entire crown, plays a larger role in light interception. The previous studies have indicated that the volume occupied by the canopy can also serve as a important indicator for measuring vegetation photosynthesis, offering valuable references for the selection of plants with enhanced photosynthetic efficiency [58]. In addition, due to the spatially explicit distribution of trees in the forest ecosystem, the influence of neighboring trees on the target tree is also considerable and cannot be overlooked [59]. Finally, although we have employed an empirical model to study the relationship between light and forest structure in order to develop a more lightweight and user-friendly tree-level model, this approach is based on research conducted on the foundation of a 3D RTM. The 3D RTM is crucial for our quantitative analysis of the light–forest structure relationship, as it possesses clear physical mechanisms and intricately depicts the light environment at the individual tree level, such a task is nearly impossible for traditional field measurements or simple models that operate at the tree level.

### Optimization theory combined with radiative transfer model

**Fig. 11.**
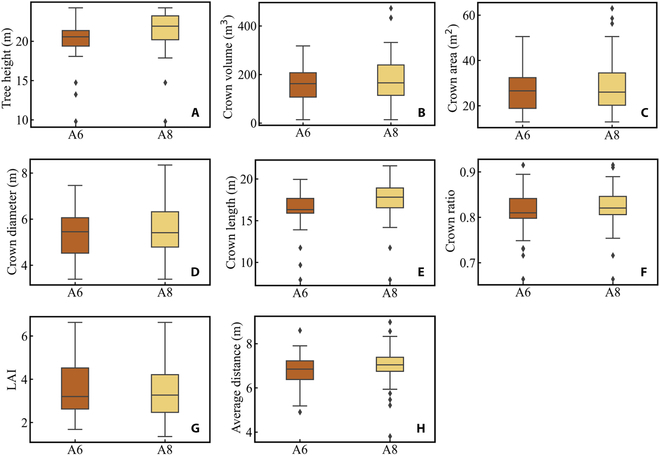
Distribution of individual tree structural parameters in A6 and A8 plots. Tree height (A), crown volume (B), crown area (C), crown diameter (D), crown length (E), crown ratio (F), LAI (G), and average distance (H).

Optimization theory is defined as the pursuit of the best and most efficient utilization of situations, opportunities, or resources. Currently, most of the research related to optimization theory in forest canopies focuses on the utilization of resources (light, water) by trees [60]. For instance, some plot-level models have also been used to estimate the photosynthetic capacity of the entire forest canopy in order to maximize its photosynthetic efficiency [61,62]. Similarly, previous studies have analyzed the impact of different loquat tree structures on light interception from the perspective of radiation transfer using a 3D loquat tree structure combined with a radiative transfer model, providing insights for cultivar breeding [63]. Canopy gap fraction and tree density are 2 important factors that impact light interception in forests. Canopy gap fraction represents the size of gaps through which light can penetrate the canopy, and a higher canopy gap fraction results in increased light penetration through the forest canopy. Consequently, it reduces the likelihood of light interception by trees. In contrast, tree density has the opposite effect on light interception. A higher tree density, accompanied by greater canopy coverage, leads to an increased probability of light interception. In our study, we primarily utilized the combination of a 3D RTM and explicit tree structures to quantitatively analyze the relationship between canopy gap fraction, tree density, and daily average cumulative APAR per tree in virtual forest scenes. The 14 larch plots showed an increasing trend followed by a decreasing trend in canopy gap fraction (Fig. 10C). The plot A2 reached its peak daily average cumulative APAR per tree, which can be attributed to its lower tree density (244 trees/ha) and larger canopy gap fraction (0.52) compared to the other 8 larch plots. Moreover, for some plots with similar canopy gap fraction and tree density (plot A6 and plot A8), there were marked differences in the average cumulative APAR per tree of the plots (Fig. 10C). This can be attributed to not only the impact of tree distribution in the stand space (Fig. 8) but also the inherent structural variations of individual tree crowns (shape and size) (Fig. 11A to G) and competition for light from surrounding neighboring trees (Fig. 11H) [64,65], which further contribute to the variations in average cumulative APAR per tree across the plot. In forest management and stand design, tree density is generally considered a more easily adjustable factor compared to canopy gap fraction. The previous research also demonstrated that thinning to reduce tree density is useful for increasing tree growth [66,67]. In our study, a natural exponential equation was used to fit the average cumulative APAR per tree and tree density. Our results also showed a significant coefficient of determination for stand density and daily-scale average cumulative APAR per tree. By controlling forest stand density, the distribution of light within the forest canopy can be effectively regulated. Simulation methods can provide insights for forest management in larch plantations, optimizing forest structure, and finding an appropriate tree density that maximizes APAR for optimal tree growth.

### Limitations of quantifying the relationship between light and forest structure

In our study, we achieved precise characterization of daily-scale individual tree APAR in larch plantations by combining a 3D RTM with explicit forest scenes. The aim was to explore the relationship between light and forest structure through simulation, providing insights for forest management practices. However, this approach only focused on the relationship between trees and light to enhance overall forest quality and carbon sequestration. Therefore, a comprehensive analysis of additional growth factors such as water and temperature are necessary. Previous research has incorporated these growth factors (light, water, and temperature) into growth models to simulate and understand vegetation growth [68,69]. Therefore, coupling the 3-dimensional radiation transfer model with growth models for simulation studies can provide more reliable data for understanding forest growth and ecological processes. Furthermore, comparative simulations of different forest structures (coniferous, broad-leaved, and mixed) would be beneficial for understanding the impact of different canopy structures on light interception. Additionally, in our individual tree reconstruction process using TLS LIDAR, the integration of leaves into branches involved the utilization of field-measured stand LAI, which was evenly distributed for each individual tree. Therefore, further attention is warranted regarding how to refine the addition of leaves based on botanical theory [70]. Finally, simulations should also consider the seasonal and annual-scale variations in light, as these long-term processes can provide fundamental data for developing light use efficiency models [71] and offer scientific references for forest management and productivity enhancement.

## Conclusion

A 3-dimensional radiative transfer model combined with LiDAR data was utilized to quantify fine-scale APAR in plantation forests. This approach enables the quantification of the relationship between the daily-scale APAR of individual trees and the forest structure. Among the 14 reconstructed virtual larch plots, the results revealed an increasing-then-decreasing trend in the average instantaneous APAR per tree of the larch forests. The peak value of the average instantaneous APAR per tree was observed at 14:00, with negligible differences between 12:00 and 14:00. A PLSR model was employed to assess the relationship between daily-scale cumulative APAR and individual tree structural parameters (crown diameter, crown area, crown length, crown ratio, crown volume, tree height, and LAI) and distance-dependent competition index. VIP scores for the 8 tree structural parameters were derived from the PLSR model. The VIP scores indicated that all 8 tree structural variables contributed to APAR to some extent. Overall, the results demonstrated that tree structural parameters related to the vertical dimension (crown ratio, tree height, and crown length) had the lowest contribution to light interception, followed by those related to the horizontal dimension (crown diameter, LAI, and crown area). Crown volume, representing the ecological niche occupied within the forest, was identified as the most important factor for light interception. In addition, the distribution of neighboring trees around each tree also has an impact on its light interception.

Furthermore, the relationship between forest structure (canopy gap fraction and tree density) and the daily-scale average cumulative APAR per tree of the forest plots was also quantified. The results showed that among the 14 forest plots, the daily-scale average cumulative APAR per tree of the plots initially increased and then decreased with canopy gap fraction, reaching its peak at a canopy gap fraction of 0.52. In contrast, tree density exhibited an opposite effect compared to canopy gap fraction. Furthermore, the tree density and daily-scale average cumulative APAR per tree were fitted with a natural exponential equation. This result reveals that tree density has an important impact on light interception by forest.

In conclusion, our research findings demonstrate the feasibility of using simulation methods to explore the relationship between individual tree structures and hard-to-measure light interceptions, providing potential to search optimal forest structures. The utilization of a 3D RTM combined with LiDAR data to reconstruct detailed forest scenes with spatially and structurally explicit information has provided insights for precise forest management, optimization of forest structure, and enhancement of forest carbon storage. Importantly, this process is non-destructive, controllable, and quantifiable for forest ecosystems.

## Data Availability

The reconstructed 14 virtual larch scenes can be accessed through the provided links (https://doi.org/10.5281/zenodo.10092254).
